# The feasibility of using smartphone apps as treatment components for depressed suicidal outpatients

**DOI:** 10.3389/fpsyt.2022.971046

**Published:** 2022-09-27

**Authors:** Chani Nuij, Wouter van Ballegooijen, Derek de Beurs, Remco F. P. de Winter, Renske Gilissen, Rory C. O’Connor, Jan H. Smit, Ad Kerkhof, Heleen Riper

**Affiliations:** ^1^Section of Clinical Psychology, Amsterdam Public Health Research Institute, Vrije Universiteit Amsterdam, Amsterdam, Netherlands; ^2^Department of Psychiatry, Amsterdam Public Health Research Institute, Amsterdam University Medical Centers (UMC) – Location Vrije Universiteit Amsterdam, Amsterdam, Netherlands; ^3^Department of Mental Health and Prevention, Trimbos Institute, Utrecht, Netherlands; ^4^Mental Health Institution, Mental Health Institution (GGZ) Rivierduinen, Leiden, Netherlands; ^5^Department of Research, 113 Suicide Prevention, Amsterdam, Netherlands; ^6^Suicidal Behaviour Research Laboratory, Institute of Health and Wellbeing, University of Glasgow, Glasgow, United Kingdom

**Keywords:** suicide, suicide prevention, mHealth (mobile health), apps, feasibility

## Abstract

Mental health smartphone apps could increase the safety and self-management of patients at risk of suicide, but it is still unclear whether it is feasible to integrate such apps into routine mental healthcare. This study reports on the feasibility of using a safety planning app (BackUp) and a self-monitoring app (mEMA) as components of the routine treatment of depressed outpatients with suicidal ideation. Clinicians were trained in working with both of the apps, and they invited their eligible patients with suicidal ideation for study participation. Patients used the apps for 3 months and discussed these with their clinician during treatment. Patients completed assessments at baseline (T_0_), 4 weeks (T_1_) and post-test (T_2_, 12 weeks after baseline). Both patients and clinicians also participated in telephone interviews. Feasibility was assessed in terms of *usability* (score > 70 on System Usability Scale, SUS), *acceptability* (score > 20 on Client Satisfaction Questionnaire-8, CSQ-8), and *uptake* (sufficient rates of component completion and app usage in treatment). The sample included 17 adult outpatients (52.9% male, age range 20–50 years) diagnosed with a depressive disorder and suicidal ideation at baseline. BackUp was rated by patients at above the cut-off scores for usability (SUS mean score at T_1_ 75.63 and at T_2_ 77.71) and acceptability (CSQ-8 mean score at T_1_ 23.42 and at T_2_ 23.50). mEMA was similarly rated (SUS mean score at T_1_ 75.83 and at T_2_ 76.25; CSQ-8 mean score at T_1_ 23.92 and at T_2_ 22.75). Telephone interviews with patients and clinicians confirmed the usability and acceptability. The uptake criteria were not met. Our findings suggest that mobile safety planning and mobile self-monitoring can be considered acceptable and usable as treatment components for depressed suicidal outpatients, but the integration of apps into routine treatment needs to be further explored.

## Introduction

Suicide is a major public health concern. Each year, more than 700,000 people die by suicide globally (1) and an estimated 14,000,000 people attempt to end their lives. In recent decades, considerable effort has been devoted to prevention strategies to reduce suicide rates (2). The rise of digital technologies such as mobile health (mHealth) has made a new platform for suicide prevention available. Recent estimates suggest that 66 applications (apps) in the Google Play and iOS app stores are related to suicidal thoughts and behaviours (3). Most such apps include a suicide prevention feature, and nearly a quarter of the apps have a feature for creating a safety plan.

Safety planning is a brief psychological intervention designed to reduce the imminent risk of suicidal behaviour by creating a set of coping strategies and sources of support in a specified plan (4, 5). During a suicidal crisis, this predetermined set of responses to an impending crisis can be used to strengthen self-help and help-seeking behaviours in order to prevent or manage suicidal urges (5). Safety planning is recommended in suicide prevention guidelines as standard care for individuals at risk of suicide (6, 7) and part of different treatment strategies like cognitive-behavioural therapy for suicide prevention (CBSP) (4, 8), and Collaborative Assessment and Management of Suicidality (CAMS) (9, 10). In the past decade, safety planning has been developed as a stand-alone intervention in the context of emergency departments, outpatient treatment or inpatient care for patients with suicidality (4, 5). A recent meta-analysis of six studies on the effectiveness of safety planning concluded that safety planning is effective in curtailing suicidal behaviour in comparison with control groups, reducing the risk of suicidal behaviour by 43% in patients who were utilising a safety plan (11). Traditionally, safety plans are completed on paper, implying that they are either carried around or stored somewhere; the safety plan could thus be inaccessible when a crisis arises. A safety plan on a person’s smartphone makes the intervention available at almost all times, thereby potentially improving the accessibility and usability of the safety plan compared with a paper-based plan. Previous studies have concluded that mobile safety planning is comfortable, easy to operate and easy to access, particularly for young people (12–15). Mental healthcare providers have reported positively on their experiences with mobile safety planning and indicated that an app would be a useful tool as part of treatment (13, 14).

mHealth apps also offer the capacity for a suicidal patient to monitor suicidal ideation and related symptoms. Self-monitoring techniques such as experience sampling or Ecological Momentary Assessment (EMA) (16) are currently being used to monitor psychological processes in real time and in the natural environments of the respondents, using self-report assessments repeated daily. These daily assessments generate information about the daily real-time course of symptoms. A recent meta-analysis of 35 studies on EMA and suicidal thoughts and behaviour concluded that EMA is generally feasible and well accepted by suicidal patients (17). EMA research using apps has found that suicidal ideation and its risk factors may fluctuate considerably over the course of hours (18). Indeed, in view of such rapid fluctuations, the study of suicidality can particularly benefit from tracking and monitoring the dynamic characteristics of the suicidal process in real time (17, 18). Furthermore, self-monitoring may serve as a feedback mechanism, giving individuals insight into the nature and dynamics of their own symptoms. This may engender feelings of control and empowerment in relation to their symptoms (19–22).

The use of mobile devices for providing support and therapy for people at risk of suicide is recommended by the World Health Organization (23). Delivery of suicide prevention interventions through mHealth apps provides many opportunities, as apps are easily accessible and can deliver support in real time and *in situ*, when individuals need help the most (24). However, it still remains unclear whether using apps as an add-on to the treatment of suicidal patients is feasible for clinicians and for the patients themselves. Healthcare professionals are often sceptical of using new technology, possibly because they are uncertain if it will work for them or benefit their patients. To overcome this barrier, end users of mHealth ought to be involved in the research process (25).

The above-mentioned potentials and pitfalls underpin our research in the Continuous Assessment for Suicide Prevention and Research (CASPAR) study. Within that study, we assessed the safety planning app BackUp and the self-monitoring app mEMA in clinical settings, querying both patients and clinicians. The current paper reports on the primary aim of the CASPAR study: an assessment of the feasibility of using BackUp and mEMA as components of routine treatment for depressed outpatients at risk of suicide.

## Materials and methods

### Study design

A single-group study design was applied, including a baseline assessment (T_0_), a second assessment at 4 weeks (T_1_), and a post-test assessment 12 weeks after baseline (T_2_). Full details of the study design can be found in the protocol paper (26). Ethical approval was obtained from the medical ethics board of the Amsterdam University Medical Centre, Location VUMC (METc number 2017.512/NL62795.029.17).

### Participants

In this study, both clinicians and patients participated. Clinicians could take part if they (1) were working at a participating Dutch specialised mental health centre, (2) were treating patients for a depressive disorder who had suicidal ideation, and (3) were willing to undergo training. There were no exclusion criteria for clinicians.

Inclusion criteria for patients were (1) being an adult outpatient aged ≥ 18, (2) having a diagnosed depressive disorder or dysthymia (as a primary or comorbid disorder), (3) having current suicidal ideation, and (4) possessing a smartphone (Android or iOS). Patients were excluded if they had insufficient competence in Dutch, had current psychotic symptoms as assessed by their clinician, or were not willing or able to use the smartphone apps.

### Sample size

There is no gold standard for calculating the sample size for a feasibility study, as the purpose of the study determines the sample size justification. In a systematic assessment of sample sizes in pilot and feasibility trials, Billingham et al. (27) reported a median sample size of 36 participants, with a range of 10–300 participants. The aim of the CASPAR study was to evaluate the feasibility of using the aforementioned two apps as treatment components. According to clinician advice, a sample of 60 patients would be feasible to recruit. With an expected 25% dropout rate, a sample of 80 patients appeared adequate for the aim of this study (26). No sample size calculation for clinicians was made.

### Intervention

The BackUp safety planning app was developed by the Flemish Centre of Expertise in Suicide Prevention (VLESP) for use in Belgium (15). A version for the Netherlands was developed in cooperation with 113 Suicide Prevention (the Dutch national suicide prevention centre). BackUp was based on the original paper-and-pencil version of Stanley and Brown’s safety planning intervention (5) and contained the same six steps (warning signs, internal coping strategies, distracting activities, social support, contacting mental health professionals, and making the environment safe). The aim of BackUp was to manage a suicidal crisis and reduce the imminent risk of suicidal behaviour. See Supplementary Appendix A for more background information on BackUp.

The self-monitoring app mEMA was developed by Ilumivu^1^ and programmed by the present researchers (CN and WB) to monitor suicidal processes by means of repeated self-report items prompted acoustically throughout the day. The self-report items were based on constructs from the integrated motivational-volitional model (IMV) (28, 29), which maps theoretically the emergence of suicidal ideation and behaviour. The items were structured in two surveys: (1) a daytime survey with 12 to 14 items prompted three times a day at semi-random sampling points between 9.30 a.m. and 6.30 p.m. with a 15-min time window for answering; and (2) an evening survey with 10–11 items that could be completed between 7.30 p.m. and 12.00 midnight, prompted once at 9.00 p.m. In addition to the two surveys, mEMA generated a graph from the daytime surveys showing the course of suicidal symptoms. The graph was viewable only on the participant’s smartphone; clinicians had no direct insight into the data. In sum, the purpose of the mEMA app was to monitor suicidal symptoms on a daily basis and provide the patient and clinician insight into the patient’s symptoms through a graph. See Supplementary Appendix B for more background information.

### Procedure

#### Clinicians

Clinicians from the three participating mental health institutions were invited by the research team to take part. Interested clinicians received a 1-h training session on how to use the apps as treatment components. The training consisted of background information, an explanation of the two apps and how they could be used in treatment. The clinicians also received a manual on how to use the apps. After the training, clinicians invited their own patients for study participation, on the basis of the inclusion criteria and the clinician’s assessment of the patient. Once a patient was approved for study inclusion, the clinician integrated the apps into the treatment from that point on. Clinicians were contacted every month by the researchers (CN or research assistants) for an update, preferably by telephone and otherwise by email. In this monthly update interview lasting 5–10 min, clinicians informed the researchers about the enrolment of patients, explained how the apps were being used in the treatment and provided feedback on the feasibility of the apps.

#### Patients

Patients were informed about the study by their clinician. Interested patients were contacted by telephone by the researchers (CN or research assistants), who explained the study and checked the inclusion criteria. If the patient met the criteria and was willing to take part, the baseline assessment (T_0_) was scheduled. During this face-to-face meeting with a researcher (CN or research assistants), the patient signed the informed consent form and completed multiple questionnaires on a computer. The researcher installed BackUp and mEMA on the patient’s smartphone and explained the apps. After the meeting, patients could use the apps immediately.

Patients completed online questionnaires on their own computer at T_1_ and T_2_. Interviews by phone were conducted after baseline, namely, at 2 days, at 14 days, and at 28, 60, and 88 days after baseline. In these interviews of 15–20 min, patients provided feedback on the feasibility of the apps. In the last interview, patients and clinicians were given the option to continue using the apps as treatment components on a voluntary basis for another 3 months.

Patients received a €5 discount code for an online mail order firm after every completed assessment (T_0_, T_1_, T_2_), as well as after completing 60% of the self-report questions during the first month after inclusion, with a maximum recompense of €20.

### Measures

#### Feasibility measures

Feasibility was defined as the extent to which the apps could be successfully used (30) by depressed suicidal outpatients and by their clinician as components of standard treatment. Feasibility was assessed in terms of usability, acceptability and uptake of the apps. Quantitative data from the patients were collected at T_1_ and T_2_; qualitative data from patients and clinicians were collected in the interviews. The questionnaires and interviews were administered multiple times to identify possible differences in outcomes over the course of the study.

##### Usability

Usability was defined as the user’s experience of effectively performing tasks in the apps (31). It was measured for patients using the System Usability Scale (SUS) (32). The questionnaire contains 10 items rated on a 5-point Likert-type scale, ranging from 1 (strongly disagree) to 5 (strongly agree). The total score ranges from 10 to 100, with higher scores indicating better usability. Following the recommendation of Bangor et al. (33), we considered a total score of 70 or higher acceptable. The reliability of the SUS is regarded as good (Omega = 0.91) (34, 35).

##### Acceptability

Acceptability was defined as the user’s satisfaction with the apps (30) and was measured in patients with the Client Satisfaction Questionnaire-8 (CSQ-8) (36). The CSQ-8 contains 8 items that are rated on a 4-point Likert-type scale, with the total score ranging from 8 to 32 and higher scores indicating better user satisfaction. A total score of 20 or higher indicates an acceptable satisfaction level. The reliability of the CSQ-8 is considered good (Cronbach’s alpha = 0.91) (37).

##### Uptake

Uptake was defined in terms of the patients’ and clinicians’ actions in employing the apps (30) and was determined by assessing (1) the completion rate of the safety plan in BackUp (we defined a good uptake rate as at least 75% of patients having completed the safety plan); (2) the completion rate of the mEMA surveys (we defined completers as having completed over 50% of the total number of mEMA surveys during the second and third month of the study); and (3) the usage rate of the apps in treatment, as determined by the clinicians (we defined a good uptake rate as at least 75% of clinicians having discussed the apps in the therapy at least once every 2 weeks).

#### Clinical measures

At T_0_, T_1_, and T_2_, each patient answered online questionnaires, including the Patient Health Questionnaire (PHQ) (38) and the Self-Injurious Thoughts and Behaviours Interview (SITBI) (39), whose data are reported in the current paper. Both the PHQ and the SITBI have good psychometric properties (38, 39).

### Data analysis

Descriptive statistics were used to examine the usability, acceptability and uptake of the apps. Paired samples *t*-tests were carried out to analyse whether total scores and scores on items differed between T_1_ and T_2_. IBM SPSS version 27 was used for analysis. The feasibility measurements were further explored through a summarisation of the qualitative data.

### Deviations from the study protocol

Separately from the protocol (26), the researchers added three telephone interviews (at 2, 14, and 60 days after baseline) to assess whether patients experienced problems with the apps, to solve any problems and to collect additional feedback.

The CASPAR study was scheduled to run from February 2019 to September 2020, but was terminated early in March 2020 due to the COVID-19 pandemic.

## Results

### Participants

#### Clinicians’ characteristics

From February 2019 to March 2020, clinicians from three Dutch specialised mental health centres were trained by the researchers (CN, DB, or research assistant). Not all clinicians at the centres could participate due to the high workload in Dutch mental healthcare, and that caused delays in the recruitment. A fourth centre could not start because of the pandemic-related study termination.

**FIGURE 1:**
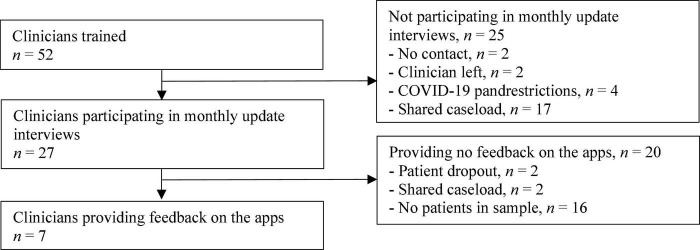
Clinicians’ enrolment flow. “Shared caseload” means that all clinicians on the team were involved in treating all patients. One clinician per team was chosen to represent the team in the study.

In the end, a total of 52 clinicians were trained, 27 of whom took part in the monthly update interviews with the researchers (see Figure 1), resulting in a total of 130 interviews. The clinicians treated fewer suicidal patients than they had estimated during the study design stage, causing a slower selection of patients for the sample. Feedback on the feasibility of the apps and information on their usage rate was ultimately provided by 7 clinicians, as those clinicians made use of the apps during the treatment. See Table 1 for the clinicians’ characteristics.

**TABLE 1:** Demographic characteristics of clinicians.

	Trained clinicians *n* = 52	Clinicians participating in interviews *n* = 27	Clinicians who provided feedback *n* = 7
**Gender**			
Male	17	6	3
Female	35	21	4
**Profession**			
Clinical psychologist or psychotherapist	2	2	0
Psychiatrist	5	2	0
Psychologist or remedial educationalist	9	7	4
Health psychologist	11	9	2
Psychiatric or other nurse	25	7	1

#### Patient study enrolment and patients’ characteristics

**FIGURE 2:**
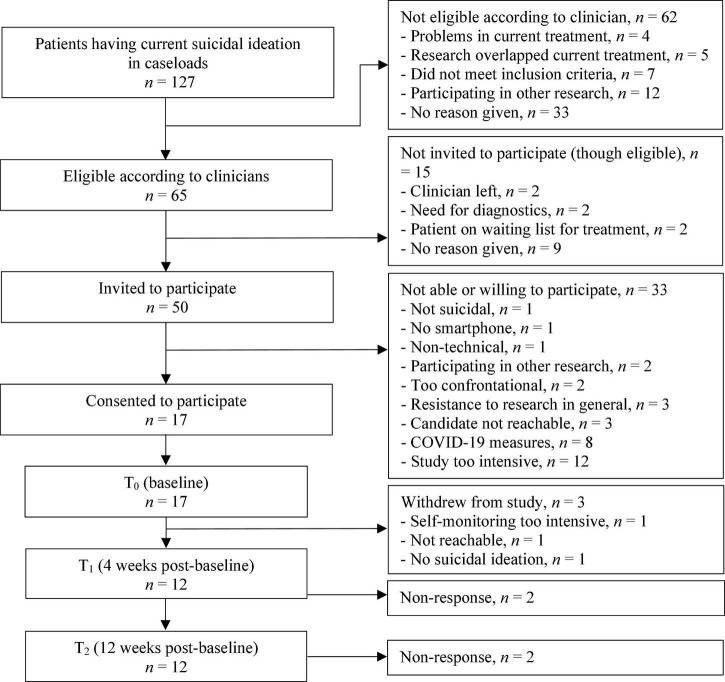
Patient recruitment flow.

From March 2019 to March 2020, 127 suicidal patients were identified by clinicians within their caseloads. Of these 127 patients, 65 were considered eligible to participate by the clinicians, and 50 were invited to participate. The main reasons for non-participation were expectations by patients that the study would be too intensive and the early termination of the study due to the COVID-19 pandemic measures. Figure 2 outlines the patient recruitment flow.

Seventeen patients aged 20–50 consented to participate. Most had a depressive disorder (*n* = 16) and one had dysthymia. Nine patients reported a lifetime suicide attempt and all patients reported recent suicidal ideation. Demographic characteristics of patients at baseline are shown in Table 2. Clinical characteristics of patients at T_0_, T_1_, and T_2_ can be found in Table 3. During the first month of the study, three patients withdrew: one was not reachable, one found that mEMA sent too many prompts, and one had a decline in suicidal symptoms which made the apps redundant.

**TABLE 2:** Demographics characteristics of patients.

	All patients *N* = 17
Age, *M (SD)*	32.12 (9.16)
**Gender, *n* (%)**	
Male	9 (52.9)
Female	8 (47.1)
**Education, *n* (%)**	
Low	6 (35.3)
Middle	6 (35.3)
High	5 (29.4)
**Employment status, *n* (%)**	
Student	4 (23.5)
Employed	7 (41.2)
Unemployed	6 (35.3)
**Living situation, *n* (%)**	
Living alone	7 (41.2)
Living with one or more people	10 (58.8)
**Psychiatric diagnosis, *n* (%)**	
Depressive disorder	16 (94.1)
Dysthymia	1 (5.9)
**Comorbid diagnosis, *n* (%)**	
ASD	5 (29.4)
PTSD	5 (29.4)
Anxiety	2 (11.8)
ADHD	2 (11.8)
Addiction	1 (5.9)
Somatic symptom disorder	1 (5.9)
**Specialised mental health team, *n* (%)**	
Depression team	7 (41.2)
Autism team	5 (29.4)
eHealth team	3 (17.6)
Crisis management team	2 (11.8)
**Phone type, *n* (%)**	
Apple iPhone	9 (52.9)
Android	8 (47.1)

**TABLE 3:** Clinical characteristics of patients.

	Baseline (T_0_) *n* = 17	4 weeks post-baseline (T_1_) *n* = 12	12 weeks post-baseline (T_2_) *n* = 12
Suicidal ideation, *n* (%)			
Past month	16 (94.1)	11 (91.7)	11 (91.7)^b^
Past week	13 (76.5)	9 (75.0)	9 (75.0)
Suicide attempt, *n* (%)	2 (11.8)^a^	0^a^ (0.0)	3 (25.0)^b^
PHQ, *M (SD)*	16.29 (6.29)	17.42 (5.73)	16.33 (6.65)

^a^Refers to past month. ^b^Refers to past two months. PHQ, Patient Health Questionnaire. Suicidal ideation and suicide attempts were assessed with the SITBI; the figure reflects the number of people, not the number of events. The suicide attempts were not reported to the researchers by patients or clinicians during the study.

### Feasibility outcomes for the BackUp safety planning app

#### Patient feasibility outcomes for BackUp

Fourteen of the seventeen patients completed the safety plan in BackUp with their clinician (three dropped out of the study). With this completion rate of 82.4%, our target rate of 75% for uptake was achieved.

The SUS total scores for BackUp indicated good usability (*n* = 12; 75.63 at T_1_ and 77.71 at T_2_; see Table 4), with no significant differences between individual item mean scores (Supplementary Appendix C) or between the two measurements, *t*(11) = −0.570, *p* = 0.580. Patients were also satisfied with the app, with CSQ-8 total scores at T_1_ and T_2_ of 23.42 and 23.50 (see Table 4). No significant difference was found between the individual item mean scores on the CSQ-8 (see Supplementary Appendix D) or between T_1_ and T_2_, *t*(11) = −0.121, *p* = 0.906.

**TABLE 4:** Patient feasibility outcomes for BackUp at T_1_ and T_2_.

	4 weeks post-baseline (T_1_) *n* = 12		12 weeks post-baseline (T_2_) *n* = 12	
	*M (SD)*	Range	*M (SD)*	Range
SUS	75.63 (10.01)	62.5–92.5	77.71 (11.40)	57.5–90.0
CSQ-8	23.42 (3.58)	16–28	23.50 (4.21)	12–28

CSQ-8, Client Satisfaction Questionnaire-8; SUS, System Usability Scale.

During the interviews, patients generally indicated that BackUp was clear and easy to use. On average, they rated the quality of BackUp as good, and most were satisfied with the amount of help they received from BackUp. The interview data further indicated that most patients used BackUp one or more times, usually to take their mind off their suicidal thoughts. However, five patients did not use the app during the study due to a decline in suicidal symptoms (*n* = 2), because they remembered the content by heart (*n* = 2), or due to resistance to using a safety plan in general, unrelated to BackUp (*n* = 1). Almost all patients indicated that it felt reassuring to be able to fall back on their safety plan if needed; that included patients who made no use of the app during the study. Having their safety plan on their smartphone, and thus always at hand, was seen as useful and convenient.

#### Clinician feasibility outcomes for BackUp

Clinicians providing feedback (*N* = 7) generally reported that completing the safety plan in BackUp with patients was easy, but some clinicians indicated that it was not practical to work together on a smartphone. Clinicians used the mobile safety plan in the same ways as a paper-and-pencil safety plan. Most clinicians discussed BackUp with their patients as needed, for example, if the client had experienced a crisis. BackUp was discussed once every 2 weeks in four patient treatments (out of twelve; usage rate 33.3%). The usage rate of 75%, which would indicate a good clinical uptake, was therefore not reached. The main reasons for not discussing BackUp every 2 weeks were a decline in suicidal symptoms (making BackUp redundant) and a lower session frequency than biweekly. However, all clinicians indicated that working with a safety plan app was suited for the standard treatment. As useful features confirming the usability of a mobile safety plan, clinicians noted that it could not easily get misplaced by the patient and that it provided interactive options (such as phoning someone from within the app). However, they pointed out that, in order to work properly using an app, the app must be embedded in the overall technological infrastructure of the institution.

### Feasibility outcomes for the mEMA self-monitoring app

#### Patient feasibility outcomes for mEMA

Patients completed a total of 2298 mEMA surveys (*N* = 17, range 3–445, *M* = 135.18, *SD* = 121.41). Three patients (out of seventeen) completed more than 50% of the surveys in the second and third month of the study, resulting in a completion rate of 17.6% (see Supplementary Appendix E). The target of 75% for uptake was therefore not reached.

The SUS scores for mEMA at T_1_ and T_2_ indicated good usability and were, respectively, 75.83 and 76.25 (see Table 5). No significant difference was found between the two measurements, *t*(11) = −0.121, *p* = 0.906, nor between individual item mean scores (see Supplementary Appendix F). The CSQ-8 total scores for mEMA indicated a good satisfaction level as well (23.92 at T_1_ and 22.75 at T_2_; see Table 5), with no significant difference between individual item mean scores (see Supplementary Appendix G) or between the two measurements, *t*(11) = 1.830, *p* = 0.095.

**TABLE 5:** Patient feasibility outcomes for mEMA at T_1_ and T_2_.

	4 weeks post-baseline (T_1_) *n* = 12		12 weeks post-baseline (T_2_) *n* = 12	
	*M (SD)*	Range	*M (SD)*	Range
SUS	75.83 (11.98)	60–97.5	76.25 (13.80)	60–95
CSQ-8	23.92 (2.99)	19–29	22.75 (3.36)	17–27

CSQ-8, Client Satisfaction Questionnaire-8; SUS, System Usability Scale.

During the interviews, patients reported that mEMA was simple, clear and user-friendly. Using mEMA was generally experienced as helpful, as patients reported that they became more aware of their suicidal symptoms through the daily monitoring and the graph. Moreover, mEMA was perceived as a useful adjunct to the treatment, as the graph helped them in discussing suicidal symptoms with their clinicians. Unfortunately, all patients experienced technical issues with mEMA, which included not receiving prompts, receiving too many prompts, and problems related to the graph (e.g., no data visible). Those issues affected the usability and clinical uptake of mEMA. In addition, two patients noted that the surveys were sometimes confrontational or irritating, and some patients felt fatigued by the repeated surveys, affecting their compliance. However, no iatrogenic effects of the self-monitoring were reported by the patients.

#### Clinician feasibility outcomes mEMA

All clinicians providing feedback (*N* = 7) used the graph in mEMA to gain information about the severity and course of suicidal symptoms. Suicidal symptoms were then discussed in a more structured way and clinicians reported that the visual outline of the symptoms made these more tangible for patients.

All clinicians indicated that mobile self-monitoring was suited for their standard treatment, as it made patients more responsible for their own treatment and the graph provided a modern, visual way to discuss symptoms. However, due to technical issues with mEMA, the graph could not be fully employed in some patient treatments. mEMA was discussed at least once every 2 weeks in three of twelve treatments (25.0%). The targeted usage rate of 75%, which would indicate a good clinical uptake, was hence not achieved.

## Discussion

This paper described the feasibility of using a safety planning app and a daily self-monitoring app as components of standard outpatient treatment for depressed adults at risk of suicide. Overall, the BackUp safety planning app and the mEMA self-monitoring app were considered usable and acceptable by patients and clinicians, as indicated by questionnaire and interview results. However, the apps were not discussed at least biweekly during treatment sessions, so the target clinical uptake of the apps was not achieved. In sum, using mobile safety planning and mobile self-monitoring as components of routine outpatient treatment appears to be usable and acceptable, but integrating the apps into treatment procedures needs to be improved.

### Mobile safety planning

According to patients and clinicians, a mobile safety plan ensures that the intervention is on hand when needed. These results are in line with previous research suggesting that mobile safety planning improves the accessibility of the safety plan and that it can be a useful tool as part of psychological treatment (12–15). The patients in the current study used BackUp mainly to avert their suicidal thoughts, which confirms the results of Pauwels et al. (15). However, a third of the patients did not use BackUp, mostly because they experienced a decrease in suicidal symptoms and hence did not need to forestall suicidal thoughts to stop a crisis. Nevertheless, such patients reported that it was reassuring to have their safety plan on their smartphone in case suicidality increased.

### Mobile self-monitoring

Information about suicidal symptoms and symptom development in mEMA was seen as insightful and helpful to signal any change of suicidal symptoms. It was suggested by patients and clinicians that mEMA led to a better awareness of suicidal symptoms, and most patients indicated that using mEMA helped them to deal with their problems more effectively. These findings provide supporting evidence for the proposed feedback mechanism of self-monitoring, which may engender feelings of control and empowerment in relation to the monitored symptoms (19–22). This is also in accordance with Sedano-Capdevila et al. (17), who suggested that EMA can potentially be a useful tool in clinical practice.

During the study, some patients felt fatigued by the repeated surveys, as was also found in previous research (17). However, the current study found no indication for iatrogenic effects of repeated daily self-monitoring on suicidal symptoms, in accordance with Coppersmith et al. (40).

### Strengths and limitations

This study is among the first to assess the feasibility of integrating apps as components of standard treatment of depressed suicidal outpatients, and one of the first to use mobile self-monitoring that provides continuous insight into the patient’s own data in the treatment context. By assessing both of the end-user groups of the apps–suicidal patients and their clinicians–we gained insight into the feasibility of using the apps in routine practice (25). In addition, we explored the outcomes of those assessments in interviews, in order to obtain a broader perspective and understanding of the usability, acceptability and uptake of the two apps in routine care.

Several limitations should be considered as well. The expected sample size was not reached due to an early termination due to the COVID-19 pandemic measures and recruitment challenges, such as the lack of suicidal patients in clinicians’ caseloads. Secondly, there may have been a selection bias in favour of patients and clinicians with an interest in apps. In addition, the quality of the safety plans and the use of apps in treatments may have been heterogeneous. Furthermore, mEMA was not used by all patients and clinicians, partly because of many technical, often software-related problems that affected the usability of the app. The results provide but a first indication, and caution is warranted about generalising the study results.

### Clinical and research implications

The results of this study suggest that mobile safety planning and mobile self-monitoring may become useful tools in clinical practice for suicidal patients. A mobile safety plan makes the intervention more accessible and offers interactive functions that can potentially have positive effects on suicidal symptoms. Currently, a Danish RCT study is assessing whether mobile safety planning is more effective in reducing suicidal symptoms as compared with a safety plan on paper (41).

Mobile self-monitoring, and insights into the stored data, can improve awareness of suicidal symptoms for both patients and clinicians. A self-monitoring app could thus be used for early detection of changes in suicidal risk factors, making it possible to act promptly on symptom deterioration and potentially to prevent suicidal behaviour. However, to make meaningful predictions of symptom deterioration, future research should examine the development, course and interaction of suicidal symptoms. Studies on the effectiveness of self-monitoring with respect to suicidal symptoms are also needed.

This study further shows that the integration of mHealth into existing treatment procedures remains a challenge, due to the novelty of apps and their initial implementation in routine care. To improve the clinical uptake of mHealth, apps have to be robust and free of technical problems. In addition, in order for apps to be usable for clinicians, apps should be reliably linked to the overall technological infrastructure of the treating institutions. Future studies on how to successfully implement mHealth in the outpatient treatment of suicidal patients are needed.

### Conclusion

Our findings tentatively support the usability and acceptability of the mobile applications, indicating that safety planning and self-monitoring apps may be a valuable addition to the treatment of suicidal patients. However, the integration of apps into existing treatment procedures needs to be improved, and more research is also needed on the effectiveness of mobile safety planning and mobile self-monitoring on suicidal symptoms.

## Data availability statement

The raw data supporting the conclusions of this article will be made available by the authors, without undue reservation.

## Ethics statement

The studies involving human participants were reviewed and approved by the Medical Ethics Board of the Amsterdam University Medical Centre, Location VUMC (METc number 2017.512/NL62795.029.17). The patients/participants provided their written informed consent to participate in this study.

## Author contributions

CN, WB, DB, RW, AK, JS, and HR were responsible for the study concept and design. CN and DB were responsible for the training of clinicians. CN contributed to the collection of the data and drafted the manuscript. CN analysed the data and discussed the results and interpretation with WB and HR. All authors critically revised the manuscript, contributed to the article, and approved the submitted version.

## Footnotes

1
https://ilumivu.com/
