# Supplementary material for: The feasibility of using smartphone apps as treatment components for depressed suicidal outpatients
Source: Front Psychiatry. 2022 Sep 27;13:971046. doi: 10.3389/fpsyt.2022.971046 (PMC9552877; doi:10.3389/fpsyt.2022.971046)
Supplement: Supplementary file 1 [file Data_Sheet_1.pdf]

frontiers

Supplementary Material

APPENDIX A: Background information on the BackUp app

BackUp was developed by the Flemish Centre of Expertise in Suicide Prevention (VLESP) in Belgium and contains multiple self-help tools for managing a suicidal crisis. Among these is a list of relevant
contact persons; a virtual hope box containing reasons for living and coping cards to help identify coping strategies; and a safety plan based on the Safety Planning Intervention (Stanley & Brown, 2012) .

An expert panel and a panel of suicidal individuals rated BackUp as valuable for suicide prevention, based on indications that the app could help users in dealing with suicidal thoughts and coping with an upcoming suicidal crisis (Pauwels et al., 2017). For use in the Netherlands, minor changes were made regarding the interface and language in collaboration with 113 Suicide Prevention, the Dutch nationwide suicide prevention helpline. This Netherlands version was used in the CASPAR study.

During the CASPAR study, feedback on the BackUp app was requested from clinicians and patients. Both reported that it would be helpful if the safety plan could be digitally exported from the app to a PDF file, in order to share the plan with a clinician or relatives. After discussion of that feedback with 113 Suicide Prevention and VLESP, a PDF export function for the safety plan was developed.

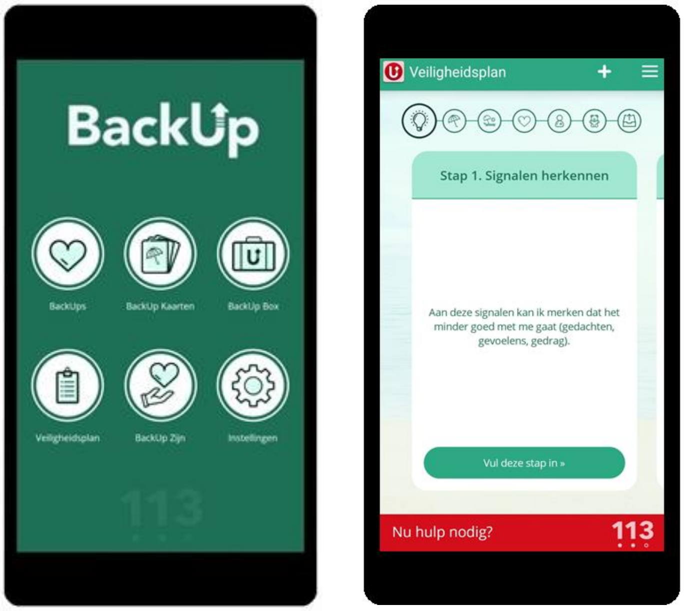

(a) (b)

Figure 3. Example of BackUp screens: (a) home screen and (b) constructing the safety plan

APPENDIX B: Background information on the mEMA app

mEMA was developed by Ilumivu (https://ilumivu.com/) and runs on both Android and iPhone smartphones. Ilumivu provided software that was programmed and customised by the researchers to monitor suicidal processes using self-report items repeated daily.

The self-report items were based on constructs from the integrated motivational-volitional model (IMV) (O'Connor, 2011; O'Connor & Kirtley, 2018), which maps theoretically the transition from thinking about suicide to acting on those thoughts. The constructs used in the present study are agitation, anxiety, burdensomeness, defeat, depressive mood, entrapment, future thoughts, hopelessness, imagery, impulsivity, problem-solving, rumination, suicidal behaviour, and suicidal ideation. For each construct, the researchers selected established retrospective questionnaires that assessed the construct. On the basis of factor analysis and face validity of the items, one item for each suicidal construct was selected and re-written in a more immediate (momentary) form (e.g. 'I feel hopeless'). The items were rated on a Likert-type scale ranging from 1 (completely disagree) to 7 (completely agree), with a neutral option 4 (neither disagree nor agree).

To limit the number of questions, two different ecological momentary assessment (EMA) surveys were developed: a daytime survey that contained constructs that were expected to fluctuate within the day, and an evening survey with constructs that were expected to fluctuate from day to day. The daytime survey contained 12 to 14 items (some follow-up questions were asked in response to answers) and was to be completed 3 times a day at semi-random sampling points between 9.30 AM and 6.30 PM, with a 15-minute time window for answering. Once per day at 9.00 PM, the patient was prompted for the evening survey, which contained 10 to 11 items (with follow-up questions in response to answers). Patients could also actively start the evening survey between 7.30 PM and 12.00 midnight. See Tables 1 and 2 for the daytime and the evening survey items.

The mEMA app also had a function that created a graph of the daytime survey variables. The graph showed the course of suicidal symptoms over a period of a day, week or month, and individual symptoms could be selected to display in the graph. See Figure 4 for screenshots of the mEMA app.

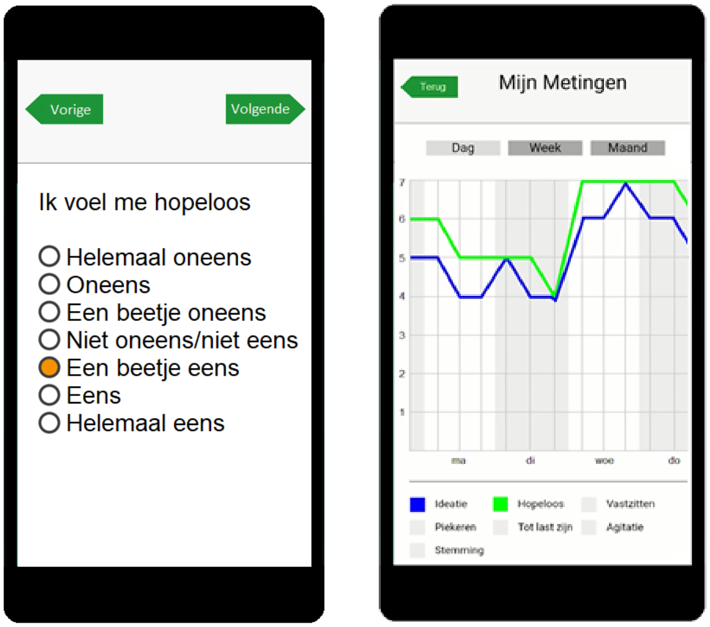

(a) (b)

Figure 4. Example of mEMA screens: (a) a survey item and (b) a graph with two selected constructs

Table 6.
mEMA daytime surveys

Item	Scale	Questionnaire	Construct
I feel depressed.	Likert	PHQ-8	Depression
I feel defeated.	Likert	D&A scale	Defeat
I feel cheerful.	Likert	HADS	Positive affect
I feel restless.	Likert	GAD-7	Anxiety
I feel trapped.	Likert	D&A scale	Entrapment
I feel satisfied.	Likert	PHQ-8	Depression
I can’t escape my thoughts.	Likert	RSS	Rumination
I’m a burden to others.	Likert	INQ-10	Burdensomeness
I have the desire to end my life.	Likert	BSS	Ideation
(if yes) I can stop thinking about killing myself.	Likert	SITBI	Ideation control
During the past hour, mental images related to suicide came to mind.	Yes / no	Flash-forwards Interview	Imagery
(if yes) How intense were those images?	Not at all intense – very intense (slider)	Flash-forwards Interview	Imagery
What applies to you now?	1. I am alone<br>2. I am with people I know<br>3. I am around people I don’t know<br>4. I am with a pet		Context

Note. Like rt scale = (1) strongly disagree, (2) disagree, (3) slightly disagree, (4) neither disagree nor agree, (5) slightly agree, (6) agree, (7) strongly agree.

Table 7.
mEMA evening survey

Item	Scale	Questionnaire	Construct
Today I felt depressed.	Likert	PHQ-8	Depression
Today I was tense.	Likert	GAD-7	Anxiety
Today I was stressed.	Likert	GAD-7	Anxiety
Today I acted without thinking.	Likert	UPPS-P	Impulsiveness
Today I felt capable of solving my problems.	Likert	UCL	Coping
Today I felt close to other people.	Likert	INQ-10	Belongingness
I wish today had gone better.	Likert	RRS	Rumination
Today I harmed myself.	Yes/no	SITBI	Self harm
(if yes) I had the intention to end my life.	Yes/no	SITBI	Suicidal self-harm
I’m looking forward to tomorrow.	Likert	RFL	Future

Note. Likert scale = (1) strongly disagree, (2) disagree, (3) slightly disagree, (4) neither disagree nor agree, (5) slightly agree, (6) agree, (7) strongly agree.

APPENDIX C: BackUp SUS results

Table 8.

Percentages of responses to SUS items at T_1 (n = 12) and T_2 (n = 12) for the BackUp app

	1: Strongly disagree (%)		2 (%)		3 (%)		4 (%)		5: Strongly agree (%)		Mean *(SD)*	
	T_1	T_2	T_1	T_2	T_1	T_2	T_1	T_2	T_1	T_2	T_1	T_2
1) I think I would like to use the BackUp app regularly.	16.7	16.7	25.0	16.7	41.7	33.3	8.3	33.3	8.3	0	2.67 (1.16)	2.83 (1.12)
2) I found the BackUp app unnecessarily complex.	50.0	66.7	41.7	33.3	8.3	0	0	0	0	0	1.58 (0.67)	1.33 (0.49)
3) I thought the BackUp app was easy to use.	0	0	8.3	0	8.3	0	58.3	66.7	25.0	33.3	4.00 (0.85)	4.33 (0.49)
4) I think I would need the support of a technical person to be able to use the BackUp app.	91.7	75.0	8.3	16.7	0	8.3	0	0	0	0	1.08 (0.29)	1.33 (0.65)
5) I found the various functions in the BackUp app were well integrated.	0	0	8.3	0	33.3	25.0	50.0	75.0	8.3	0	3.58 (0.79)	3.75 (0.45)
6) I thought there was too much inconsistency in the BackUp app.	50.0	50.0	25.0	16.7	16.7	33.3	8.3	0	0	0	1.83 (1.03)	1.83 (0.94)
7) I would imagine that most people would learn to use the BackUp app very quickly.	0	0	0	0	16.7	8.3	66.7	41.7	16.7	50.0	4.00 (0.60)	4.42 (0.67)
8) I found the BackUp app very cumbersome to use.	66.7	58.3	16.7	33.3	16.7	8.3	0	0	0	0	1.50 (0.80)	1.50 (0.67)
9) I felt very confident using the BackUp app.	8.3	8.3	0	8.3	33.3	33.3	50.0	41.7	8.3	8.3	3.50 (1.00)	3.33 (1.07)
10) I needed to learn a lot of things before I could get going with the BackUp app.	50.0	50.0	50.0	41.7	0	8.3	0	0	0	0	1.50 (0.52)	1.58 (0.67)

Note. For odd-numbered questions, greater values (4, 5) indicate better usability. For even-numbered questions, lower values (1, 2) indicate better usability.

APPENDIX D: BackUp CSQ-8 results

Table 9.

Percentages of responses to CSQ-8 items at T_1 (n = 12) and T_2 (n = 12) for the BackUp app

	1 (%)		2 (%)		3 (%)		4 (%)		Mean (*SD*)	
	T_1	T_2	T_1	T_2	T_1	T_2	T_1	T_2	T_1	T_2
1) How would you rate the quality of the service you received?	Poor		Fair		Good		Excellent		2.83 (0.39)	3.00 (0.43)
	0	0	16.7	8.3	83.3	83.3	0	8.3		
2) Did you get the kind of service you wanted?	No definitely not		No not at all		Yes generally		Yes definitely		2.83 (0.58)	2.75 (0.62)
	0	8.3	25.0	8.3	66.7	83.3	8.3	0		
3) To what extent has the BackUp app met your needs?	None met		Only a few met		Most met		Almost all met		2.83 (0.94)	2.83 (0.84)
	8.3	8.3	25.0	16.7	41.7	58.3	25.0	16.7		
4) If a friend were in need of similar help, would you recommend the BackUp app?	Definitely not		No I do not think so		Yes I think so		Yes definitely		3.08 (0.52)	3.17 (0.58)
	0	0	8.3	8.3	75.0	66.7	16.7	25.0		
5) How satisfied are you with the amount of help you received?	Quite dissatisfied		Indifferent		Mostly satisfied		Very satisfied		3.00 (0.0)	3.00 (0.74)
	0	8.3	0	0	100.0	75.0	0	16.7		
6) Has the BackUp app helped you deal more effectively with your problems?	No made it worse		No did not help		Yes somewhat		Yes a great deal		2.92 (0.79)	2.83 (0.58)
	0	0	33.3	25.0	41.7	66.7	25.0	8.3		
7) In an overall sense, how satisfied are you with the BackUp app?	Quite dissatisfied		Indifferent		Mostly satisfied		Very satisfied		3.00 (0.60)	3.08 (0.52)
	0	0	16.7	8.3	66.7	75.0	16.7	16.7		
8) If you were seeking help again, would you use the BackUp app again?	No definitely not		No I do not think so		Yes I think so		Yes definitely		2.92 (0.67)	2.83 (0.72)
	0	8.3	25.0	8.3	58.3	75.0	16.7	8.3		

APPENDIX E: mEMA surveys

Table 10.
Completed mEMA surveys

Patient	Total completed surveys in the study	Completed surveys in months 2 and 3 (%)*
1	445	167 (68.7)
2	231	121 (49.8)
3	80	16 (6.6)
4	255	163 (67.1)
5[a]	13	0 (0.0)
6	90	58 (23.9)
7	124	44 (18.1)
8	275	51 (21.0)
9	19	1 (0.4)
10 [a]	3	0 (0.0)
11 [a]	5	0 (0.0)
12	82	6 (2.5)
13	155	48 (29.8)
14	110	37 (15.2)
15	105	40 (16.5)
16	45	5 (2.1)
17	261	164 (67.5)

Note. [a] Study dropout.
* Percentage represents the proportion of daily surveys answered relative to the total number of possible surveys in months 2 and 3 (N = 243 surveys).

APPENDIX F: mEMA SUS results

Table 11.

Percentages of responses to SUS items at T_1 (n = 12) and T_2 (n = 12) for the mEMA app

	1: Strongly disagree (%)		2 (%)		3 (%)		4 (%)		5: Strongly agree (%)		Mean *(SD)*	
	T_1	T_2	T_1	T_2	T_1	T_2	T_1	T_2	T_1	T_2	T_1	T_2
1) I think I would like to use the mEMA app regularly.	0	0	8.3	16.7	41.7	25.0	41.7	50.0	8.3	8.3	3.50 (0.80)	3.50 (0.91)
2) I found the mEMA app unnecessarily complex.	41.7	33.3	41.7	58.3	16.7	8.3	0	0	0	0	1.75 (0.75)	1.75 (0.62)
3) I thought the mEMA app was easy to use.	0	0	0	0	8.3	8.3	75.0	58.3	16.7	33.3	4.08 (0.52)	4.25 (0.62)
4) I think I would need the support of a technical person to be able to use the mEMA app.	66.7	50.0	16.7	33.3	0	8.3	0	8.3	16.7	0	1.83 (1.53)	1.75 (0.97)
5) I found the various functions in the mEMA app were well integrated.	0	0	25.0	0	41.7	66.7	25.0	25.0	8.3	8.3	3.17 (0.94)	3.42 (0.67)
6) I thought there was too much inconsistency in the mEMA app.	41.7	33.3	50.0	66.7	8.3	0	0	0	0	0	1.67 (0.65)	1.67 (0.49)
7) I would imagine that most people would learn to use the mEMA app very quickly.	0	0	0	0	25.0	16.7	66.7	66.7	8.3	16.7	3.83 (0.58)	4.00 (0.60)
8) I found the mEMA app very cumbersome to use.	58.3	50.0	41.7	25.0	0	25.0	0	0	0	0	1.42 (0.52)	1.75 (0.87)
9) I felt very confident using the mEMA app.	0	0	0	0	41.7	41.7	41.7	33.3	16.7	25.0	3.75 (0.75)	3.83 (0.84)
10) I needed to learn a lot of things before I could get going with the mEMA app.	75.0	50.0	16.7	41.7	8.3	8.3	0	0	0	0	1.33 (0.65)	1.58 (0.67)

Note. For odd-numbered questions, greater values (4, 5) indicate better usability. For even-numbered questions, lower values (1, 2) indicate better usability.

APPENDIX G: mEMA CSQ-8 results

Table 12.

Percentages of responses to CSQ-8 items at T_1 (n = 12) and T_2 (n = 12) for the mEMA app

	1 (%)		2 (%)		3 (%)		4 (%)		Mean *(SD)*	
	T_1	T_2	T_1	T_2	T_1	T_2	T_1	T_2	T_1	T_2
1) How would you rate the quality of the service you received?	Poor		Fair		Good		Excellent		2.92 (0.29)	2.75 (0.45)
	0	0	8.3	25.0	91.7	75.0	0	0		
2) Did you get the kind of service you wanted?	No definitely not		No not at all		Yes generally		Yes definitely		3.17 (0.39)	2.83 (0.72)
	0	8.3	0	8.3	83.3	75.0	16.7	8.3		
3) To what extent has the mEMA app met your needs?	None met		Only a few met		Most met		Almost all met		2.75 (0.62)	2.75 (0.62)
	0	0	33.3	33.3	58.3	58.3	8.3	8.3		
4) If a friend were in need of similar help, would you recommend the mEMA app?	Definitely not		No I do not think so		Yes I think so		Yes definitely		3.17 (0.58)	3.08 (0.52)
	0	0	8.3	8.3	66.7	75.0	25.0	16.7		
5) How satisfied are you with the amount of help you received?	Quite dissatisfied		Indifferent		Mostly satisfied		Very satisfied		3.00 (0.60)	2.83 (0.58)
	0	0	16.7	25.0	66.7	66.7	16.7	8.3		
6) Has the mEMA app helped you deal more effectively with your problems?	No made it worse		No did not help		Yes somewhat		Yes a great deal		2.83 (0.58)	2.75 (0.62)
	0	0	25.0	33.3	66.7	58.3	8.3	8.3		
7) In an overall sense, how satisfied are you with the mEMA app?	Quite dissatisfied		Indifferent		Mostly satisfied		Very satisfied		3.17 (0.58)	2.92 (0.67)
	0	0	8.3	25.0	66.7	58.3	25.0	16.7		
8) If you were seeking help again, would you use the mEMA app again?	No definitely not		No I do not think so		Yes I think so		Yes definitely		2.92 (0.67)	2.83 (0.39)
	0	0	25.0	16.7	58.3	83.3	16.7	0		
